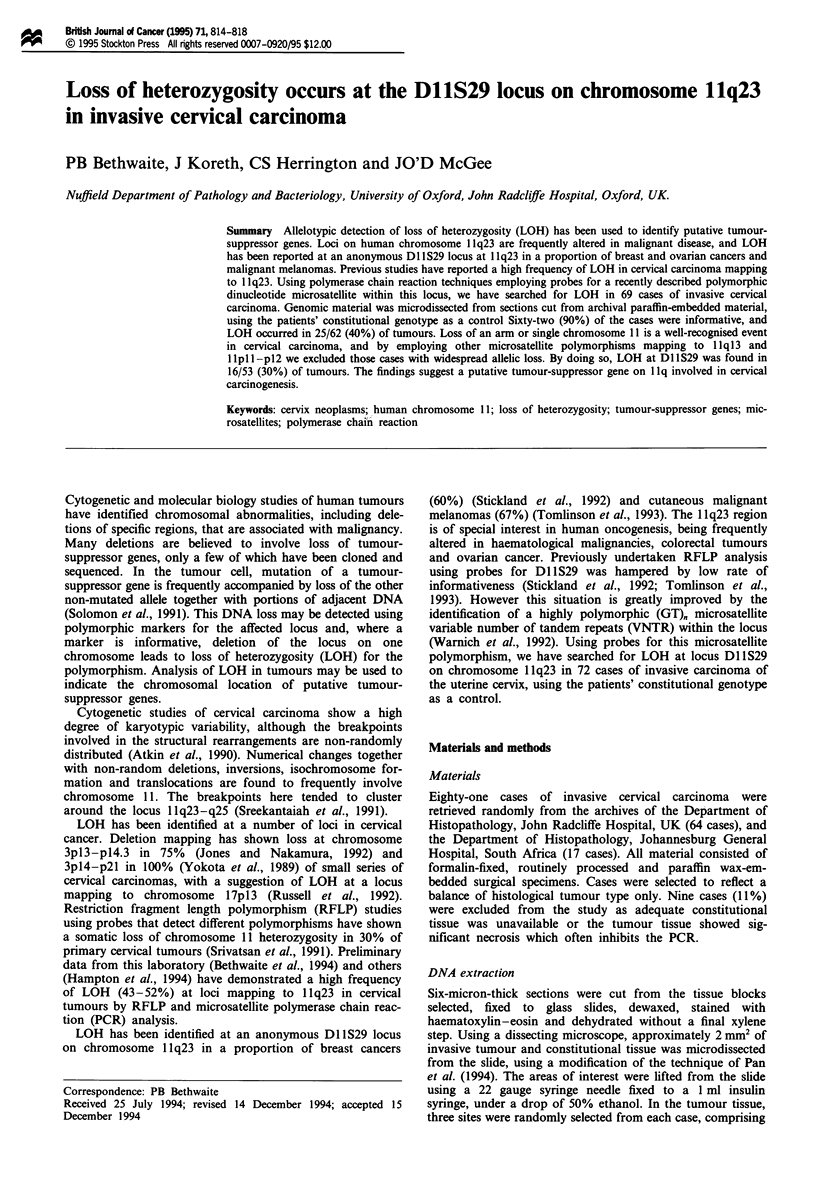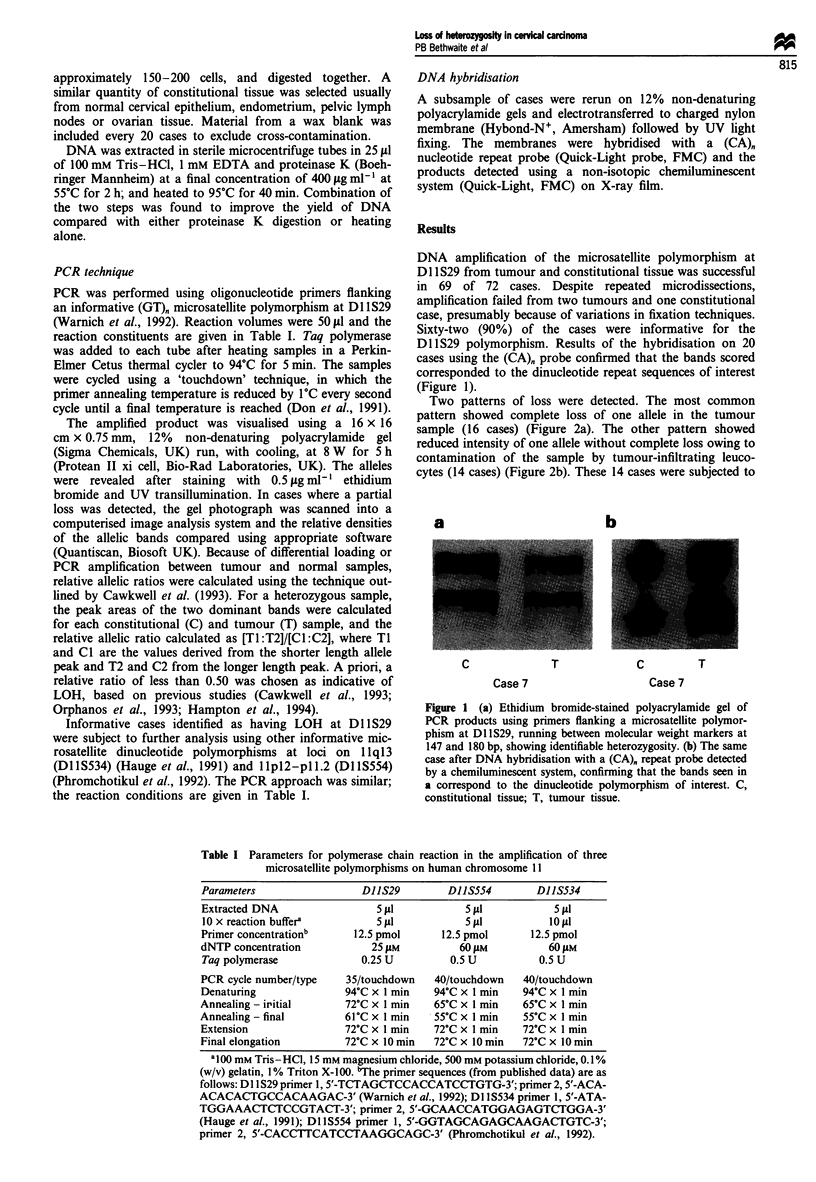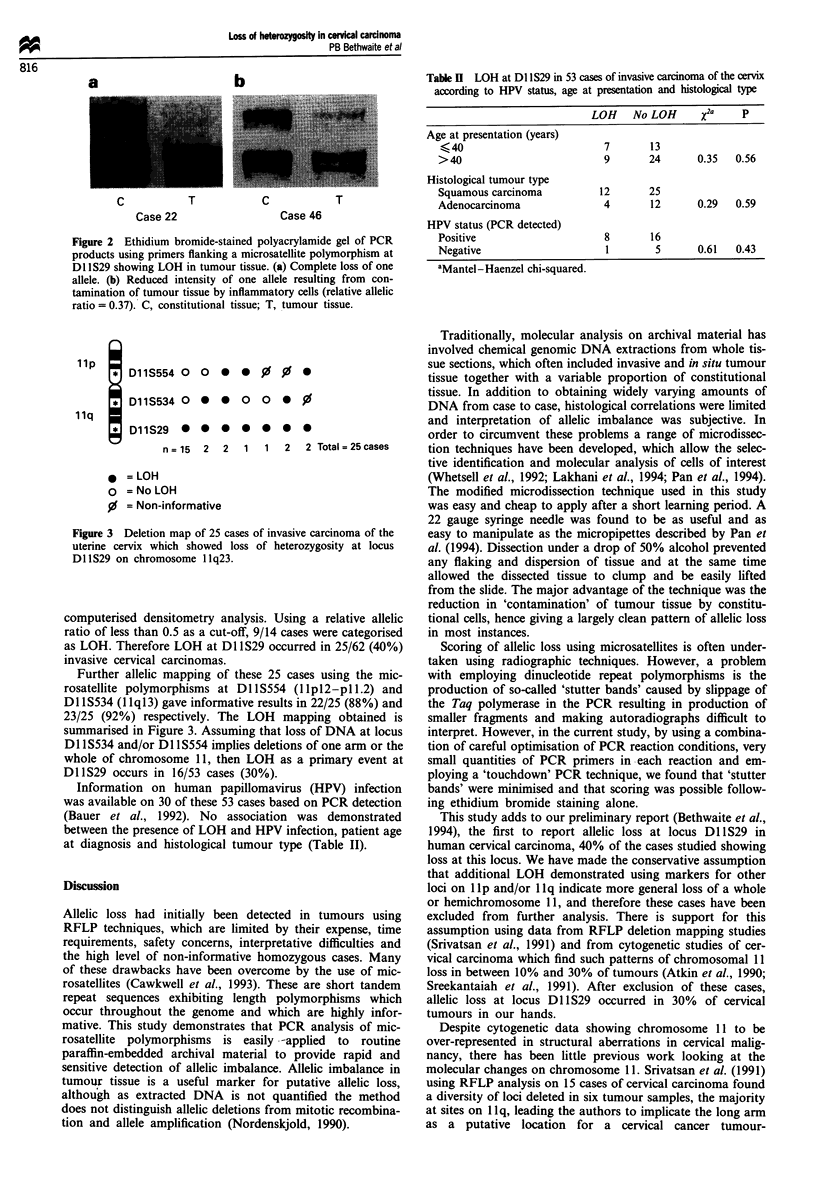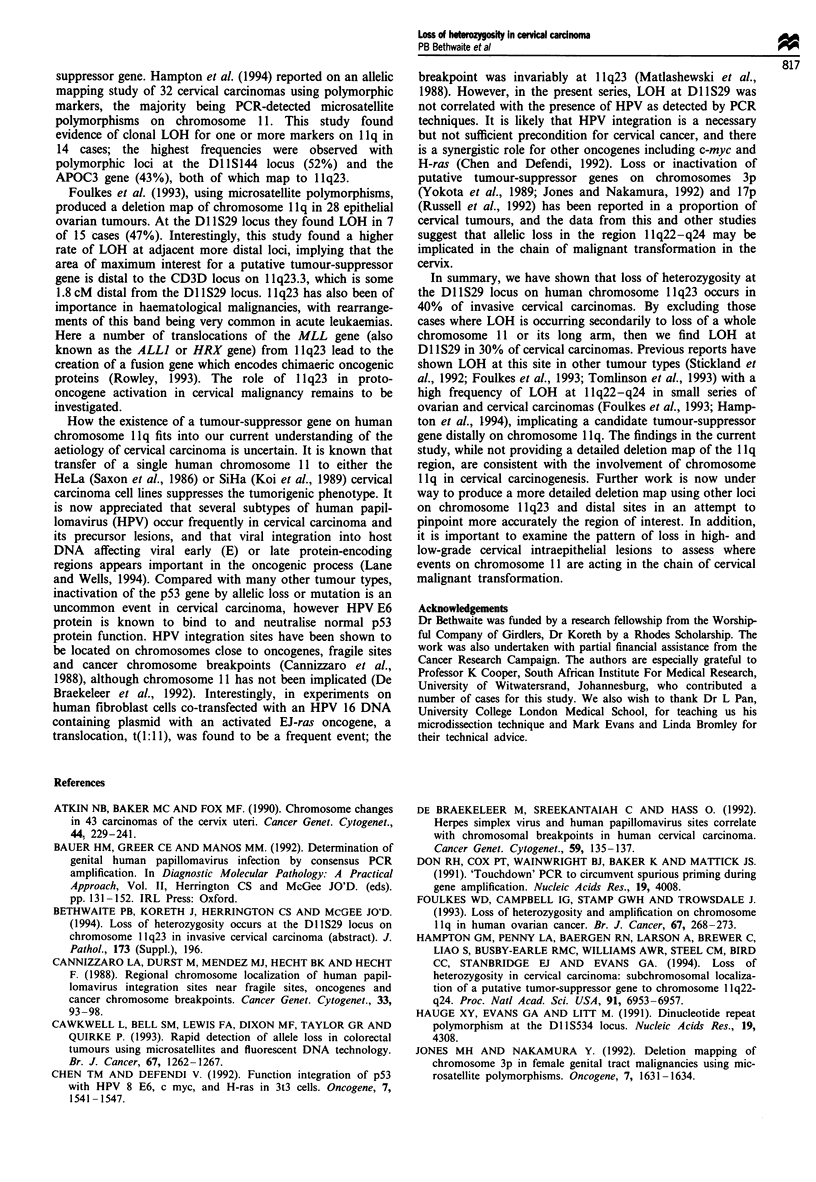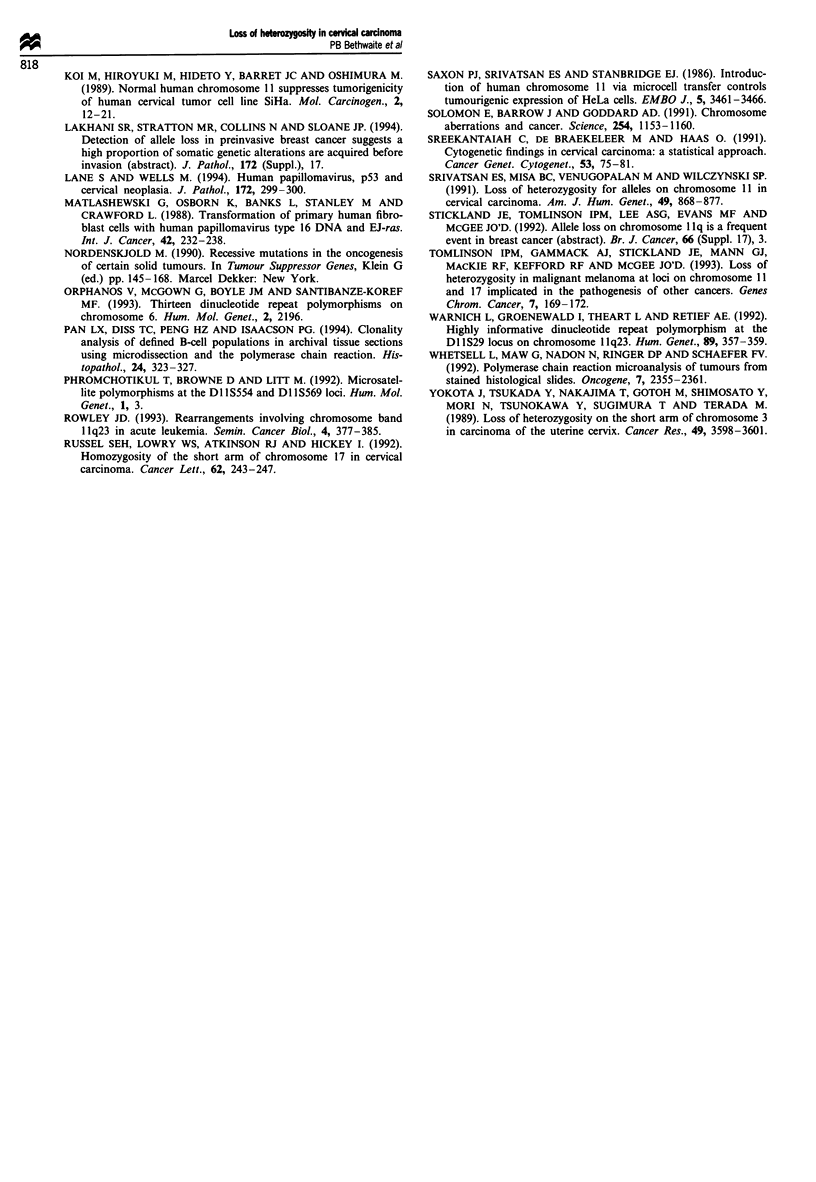# Loss of heterozygosity occurs at the D11S29 locus on chromosome 11q23 in invasive cervical carcinoma.

**DOI:** 10.1038/bjc.1995.157

**Published:** 1995-04

**Authors:** P. B. Bethwaite, J. Koreth, C. S. Herrington, J. O. McGee

**Affiliations:** Nuffield Department of Pathology and Bacteriology, University of Oxford, John Radcliffe Hospital, UK.

## Abstract

**Images:**


British Journal of Cancer (1995) 71, 814–818
© 1995 Stockton Press All rights reserved 0007-0920/95 $12.00

### Loss of heterozygosity occurs at the D11S29 locus on chromosome 11q23 in invasive cervical carcinoma

PB Bethwaite, J Koreth, CS Herrington and JO'D McGee

*Nuffield Department of Pathology and Bacteriology, University of Oxford, John Radcliffe Hospital, Oxford, UK.*

**Summary** Allelotypic detection of loss of heterozygosity (LOH) has been used to identify putative tumour-suppressor genes. Loci on human chromosome 11q23 are frequently altered in malignant disease, and LOH has been reported at an anonymous D11S29 locus at 11q23 in a proportion of breast and ovarian cancers and malignant melanomas. Previous studies have reported a high frequency of LOH in cervical carcinoma mapping to 11q23. Using polymerase chain reaction techniques employing probes for a recently described polymorphic dinucleotide microsatellite within this locus, we have searched for LOH in 69 cases of invasive cervical carcinoma. Genomic material was microdissected from sections cut from archival paraffin-embedded material, using the patients' constitutional genotype as a control Sixty-two (90%) of the cases were informative, and LOH occurred in 25/62 (40%) of tumours. Loss of an arm or single chromosome 11 is a well-recognised event in cervical carcinoma, and by employing other microsatellite polymorphisms mapping to 11q13 and 11p11–p12 we excluded those cases with widespread allelic loss. By doing so, LOH at D11S29 was found in 16/53 (30%) of tumours. The findings suggest a putative tumour-suppressor gene on 11q involved in cervical carcinogenesis.

**Keywords**: cervix neoplasms; human chromosome 11; loss of heterozygosity; tumour-suppressor genes; microsatellites; polymerase chain reaction

Cytogenetic and molecular biology studies of human tumours have identified chromosomal abnormalities, including deletions of specific regions, that are associated with malignancy. Many deletions are believed to involve loss of tumour-suppressor genes, only a few of which have been cloned and sequenced. In the tumour cell, mutation of a tumour-suppressor gene is frequently accompanied by loss of the other non-mutated allele together with portions of adjacent DNA (Solomon *et al.*, 1991). This DNA loss may be detected using polymorphic markers for the affected locus and, where a marker is informative, deletion of the locus on one chromosome leads to loss of heterozygosity (LOH) for the polymorphism. Analysis of LOH in tumours may be used to indicate the chromosomal location of putative tumour-suppressor genes.

Cytogenetic studies of cervical carcinoma show a high degree of karyotypic variability, although the breakpoints involved in the structural rearrangements are non-randomly distributed (Atkin *et al.*, 1990). Numerical changes together with non-random deletions, inversions, isochromosome formation and translocations are found to frequently involve chromosome 11. The breakpoints here tended to cluster around the locus 11q23–q25 (Sreekantaiah *et al.*, 1991).

LOH has been identified at a number of loci in cervical cancer. Deletion mapping has shown loss at chromosome 3p13–p14.3 in 75% (Jones and Nakamura, 1992) and 3p14–p21 in 100% (Yokota *et al.*, 1989) of small series of cervical carcinomas, with a suggestion of LOH at a locus mapping to chromosome 17p13 (Russell *et al.*, 1992). Restriction fragment length polymorphism (RFLP) studies using probes that detect different polymorphisms have shown a somatic loss of chromosome 11 heterozygosity in 30% of primary cervical tumours (Srivatsan *et al.*, 1991). Preliminary data from this laboratory (Bethwaite *et al.*, 1994) and others (Hampton *et al.*, 1994) have demonstrated a high frequency of LOH (43–52%) at loci mapping to 11q23 in cervical tumours by RFLP and microsatellite polymerase chain reaction (PCR) analysis.

LOH has been identified at an anonymous D11S29 locus on chromosome 11q23 in a proportion of breast cancers (60%) (Stickland *et al.*, 1992) and cutaneous malignant melanomas (67%) (Tomlinson *et al.*, 1993). The 11q23 region is of special interest in human oncogenesis, being frequently altered in haematological malignancies, colorectal tumours and ovarian cancer. Previously undertaken RFLP analysis using probes for D11S29 was hampered by low rate of informativeness (Stickland *et al.*, 1992; Tomlinson *et al.*, 1993). However this situation is greatly improved by the identification of a highly polymorphic $(GT)_n$ microsatellite variable number of tandem repeats (VNTR) within the locus (Warnich *et al.*, 1992). Using probes for this microsatellite polymorphism, we have searched for LOH at locus D11S29 on chromosome 11q23 in 72 cases of invasive carcinoma of the uterine cervix, using the patients' constitutional genotype as a control.

### Materials and methods

### Materials

Eighty-one cases of invasive cervical carcinoma were retrieved randomly from the archives of the Department of Histopathology, John Radcliffe Hospital, UK (64 cases), and the Department of Histopathology, Johannesburg General Hospital, South Africa (17 cases). All material consisted of formalin-fixed, routinely processed and paraffin wax-embedded surgical specimens. Cases were selected to reflect a balance of histological tumour type only. Nine cases (11%) were excluded from the study as adequate constitutional tissue was unavailable or the tumour tissue showed significant necrosis which often inhibits the PCR.

### DNA extraction

Six-micron-thick sections were cut from the tissue blocks selected, fixed to glass slides, dewaxed, stained with haematoxylin–eosin and dehydrated without a final xylene step. Using a dissecting microscope, approximately 2 $mm^2$ of invasive tumour and constitutional tissue was microdissected from the slide, using a modification of the technique of Pan *et al.* (1994). The areas of interest were lifted from the slide using a 22 gauge syringe needle fixed to a 1 ml insulin syringe, under a drop of 50% ethanol. In the tumour tissue, three sites were randomly selected from each case, comprising

Correspondence: PB Bethwaite
Received 25 July 1994; revised 14 December 1994; accepted 15 December 1994

approximately 150–200 cells, and digested together. A similar quantity of constitutional tissue was selected usually from normal cervical epithelium, endometrium, pelvic lymph nodes or ovarian tissue. Material from a wax blank was included every 20 cases to exclude cross-contamination.

DNA was extracted in sterile microcentrifuge tubes in 25 µl of 100 mM Tris-HCl, 1 mM EDTA and proteinase K (Boehringer Mannheim) at a final concentration of 400 $\mu g\ ml^{-1}$ at 55°C for 2 h; and heated to 95°C for 40 min. Combination of the two steps was found to improve the yield of DNA compared with either proteinase K digestion or heating alone.

*PCR technique*

PCR was performed using oligonucleotide primers flanking an informative $(GT)_n$ microsatellite polymorphism at D11S29 (Warnich *et al*., 1992). Reaction volumes were 50 µl and the reaction constituents are given in Table I. *Taq* polymerase was added to each tube after heating samples in a Perkin-Elmer Cetus thermal cycler to 94°C for 5 min. The samples were cycled using a 'touchdown' technique, in which the primer annealing temperature is reduced by 1°C every second cycle until a final temperature is reached (Don *et al*., 1991).

The amplified product was visualised using a 16 × 16 cm × 0.75 mm, 12% non-denaturing polyacrylamide gel (Sigma Chemicals, UK) run, with cooling, at 8 W for 5 h (Protean II xi cell, Bio-Rad Laboratories, UK). The alleles were revealed after staining with 0.5 $\mu g\ ml^{-1}$ ethidium bromide and UV transillumination. In cases where a partial loss was detected, the gel photograph was scanned into a computerised image analysis system and the relative densities of the allelic bands compared using appropriate software (Quantiscan, Biosoft UK). Because of differential loading or PCR amplification between tumour and normal samples, relative allelic ratios were calculated using the technique outlined by Cawkwell *et al*. (1993). For a heterozygous sample, the peak areas of the two dominant bands were calculated for each constitutional (C) and tumour (T) sample, and the relative allelic ratio calculated as [T1:T2]/[C1:C2], where T1 and C1 are the values derived from the shorter length allele peak and T2 and C2 from the longer length peak. A priori, a relative ratio of less than 0.50 was chosen as indicative of LOH, based on previous studies (Cawkwell *et al*., 1993; Orphanos *et al*., 1993; Hampton *et al*., 1994).

Informative cases identified as having LOH at D11S29 were subject to further analysis using other informative microsatellite dinucleotide polymorphisms at loci on 11q13 (D11S534) (Hauge *et al*., 1991) and 11p12–p11.2 (D11S554) (Phromchotikul *et al*., 1992). The PCR approach was similar; the reaction conditions are given in Table I.

*DNA hybridisation*

A subsample of cases were rerun on 12% non-denaturing polyacrylamide gels and electrotransferred to charged nylon membrane (Hybond-$N^+$, Amersham) followed by UV light fixing. The membranes were hybridised with a $(CA)_n$ nucleotide repeat probe (Quick-Light probe, FMC) and the products detected using a non-isotopic chemiluminescent system (Quick-Light, FMC) on X-ray film.

### Results

DNA amplification of the microsatellite polymorphism at D11S29 from tumour and constitutional tissue was successful in 69 of 72 cases. Despite repeated microdissections, amplification failed from two tumours and one constitutional case, presumably because of variations in fixation techniques. Sixty-two (90%) of the cases were informative for the D11S29 polymorphism. Results of the hybridisation on 20 cases using the $(CA)_n$ probe confirmed that the bands scored corresponded to the dinucleotide repeat sequences of interest (Figure 1).

Two patterns of loss were detected. The most common pattern showed complete loss of one allele in the tumour sample (16 cases) (Figure 2a). The other pattern showed reduced intensity of one allele without complete loss owing to contamination of the sample by tumour-infiltrating leucocytes (14 cases) (Figure 2b). These 14 cases were subjected to

**Figure 1** (**a**) Ethidium bromide-stained polyacrylamide gel of PCR products using primers flanking a microsatellite polymorphism at D11S29, running between molecular weight markers at 147 and 180 bp, showing identifiable heterozygosity. (**b**) The same case after DNA hybridisation with a $(CA)_n$ repeat probe detected by a chemiluminescent system, confirming that the bands seen in **a** correspond to the dinucleotide polymorphism of interest. C, constitutional tissue; T, tumour tissue.

**Table I** Parameters for polymerase chain reaction in the amplification of three microsatellite polymorphisms on human chromosome 11

| *Parameters* | *D11S29* | *D11S554* | *D11S534* |
|---|---|---|---|
| Extracted DNA | 5 µl | 5 µl | 5 µl |
| 10 × reaction buffer[a] | 5 µl | 5 µl | 10 µl |
| Primer concentration[b] | 12.5 pmol | 12.5 pmol | 12.5 pmol |
| dNTP concentration | 25 µM | 60 µM | 60 µM |
| *Taq* polymerase | 0.25 U | 0.5 U | 0.5 U |
| PCR cycle number/type | 35/touchdown | 40/touchdown | 40/touchdown |
| Denaturing | 94°C × 1 min | 94°C × 1 min | 94°C × 1 min |
| Annealing – initial | 72°C × 1 min | 65°C × 1 min | 65°C × 1 min |
| Annealing – final | 61°C × 1 min | 55°C × 1 min | 55°C × 1 min |
| Extension | 72°C × 1 min | 72°C × 1 min | 72°C × 1 min |
| Final elongation | 72°C × 10 min | 72°C × 10 min | 72°C × 10 min |

[a]100 mM Tris-HCl, 15 mM magnesium chloride, 500 mM potassium chloride, 0.1% (w/v) gelatin, 1% Triton X-100. [b]The primer sequences (from published data) are as follows: D11S29 primer 1, 5′-TCTAGCTCCACCATCCTGTG-3′; primer 2, 5′-ACA-ACACACTGCCACAAGAC-3′ (Warnich *et al*., 1992); D11S534 primer 1, 5′-ATA-TGGAAACTCTCCGTACT-3′; primer 2, 5′-GCAACCATGGAGAGTCTGGA-3′ (Hauge *et al*., 1991); D11S554 primer 1, 5′-GGTAGCAGAGCAAGACTGTC-3′; primer 2, 5′-CACCTTCATCCTAAGGCAGC-3′ (Phromchotikul *et al*., 1992).

**Figure 2** Ethidium bromide-stained polyacrylamide gel of PCR products using primers flanking a microsatellite polymorphism at D11S29 showing LOH in tumour tissue. (**a**) Complete loss of one allele. (**b**) Reduced intensity of one allele resulting from contamination of tumour tissue by inflammatory cells (relative allelic ratio = 0.37). C, constitutional tissue; T, tumour tissue.

**Figure 3** Deletion map of 25 cases of invasive carcinoma of the uterine cervix which showed loss of heterozygosity at locus D11S29 on chromosome 11q23.

computerised densitometry analysis. Using a relative allelic ratio of less than 0.5 as a cut-off, 9/14 cases were categorised as LOH. Therefore LOH at D11S29 occurred in 25/62 (40%) invasive cervical carcinomas.

Further allelic mapping of these 25 cases using the microsatellite polymorphisms at D11S554 (11p12–p11.2) and D11S534 (11q13) gave informative results in 22/25 (88%) and 23/25 (92%) respectively. The LOH mapping obtained is summarised in Figure 3. Assuming that loss of DNA at locus D11S534 and/or D11S554 implies deletions of one arm or the whole of chromosome 11, then LOH as a primary event at D11S29 occurs in 16/53 cases (30%).

Information on human papillomavirus (HPV) infection was available on 30 of these 53 cases based on PCR detection (Bauer *et al.*, 1992). No association was demonstrated between the presence of LOH and HPV infection, patient age at diagnosis and histological tumour type (Table II).

**Table II** LOH at D11S29 in 53 cases of invasive carcinoma of the cervix according to HPV status, age at presentation and histological type

| | *LOH* | *No LOH* | $\chi^{2a}$ | P |
|---|---|---|---|---|
| Age at presentation (years) | | | | |
| $\leqslant$40 | 7 | 13 | | |
| >40 | 9 | 24 | 0.35 | 0.56 |
| Histological tumour type | | | | |
| Squamous carcinoma | 12 | 25 | | |
| Adenocarcinoma | 4 | 12 | 0.29 | 0.59 |
| HPV status (PCR detected) | | | | |
| Positive | 8 | 16 | | |
| Negative | 1 | 5 | 0.61 | 0.43 |

[a]Mantel–Haenzel chi-squared.

### Discussion

Allelic loss had initially been detected in tumours using RFLP techniques, which are limited by their expense, time requirements, safety concerns, interpretative difficulties and the high level of non-informative homozygous cases. Many of these drawbacks have been overcome by the use of microsatellites (Cawkwell *et al.*, 1993). These are short tandem repeat sequences exhibiting length polymorphisms which occur throughout the genome and which are highly informative. This study demonstrates that PCR analysis of microsatellite polymorphisms is easily applied to routine paraffin-embedded archival material to provide rapid and sensitive detection of allelic imbalance. Allelic imbalance in tumour tissue is a useful marker for putative allelic loss, although as extracted DNA is not quantified the method does not distinguish allelic deletions from mitotic recombination and allele amplification (Nordenskjold, 1990).

Traditionally, molecular analysis on archival material has involved chemical genomic DNA extractions from whole tissue sections, which often included invasive and *in situ* tumour tissue together with a variable proportion of constitutional tissue. In addition to obtaining widely varying amounts of DNA from case to case, histological correlations were limited and interpretation of allelic imbalance was subjective. In order to circumvent these problems a range of microdissection techniques have been developed, which allow the selective identification and molecular analysis of cells of interest (Whetsell *et al.*, 1992; Lakhani *et al.*, 1994; Pan *et al.*, 1994). The modified microdissection technique used in this study was easy and cheap to apply after a short learning period. A 22 gauge syringe needle was found to be as useful and as easy to manipulate as the micropipettes described by Pan *et al.* (1994). Dissection under a drop of 50% alcohol prevented any flaking and dispersion of tissue and at the same time allowed the dissected tissue to clump and be easily lifted from the slide. The major advantage of the technique was the reduction in 'contamination' of tumour tissue by constitutional cells, hence giving a largely clean pattern of allelic loss in most instances.

Scoring of allelic loss using microsatellites is often undertaken using radiographic techniques. However, a problem with employing dinucleotide repeat polymorphisms is the production of so-called 'stutter bands' caused by slippage of the *Taq* polymerase in the PCR resulting in production of smaller fragments and making autoradiographs difficult to interpret. However, in the current study, by using a combination of careful optimisation of PCR reaction conditions, very small quantities of PCR primers in each reaction and employing a 'touchdown' PCR technique, we found that 'stutter bands' were minimised and that scoring was possible following ethidium bromide staining alone.

This study adds to our preliminary report (Bethwaite *et al.*, 1994), the first to report allelic loss at locus D11S29 in human cervical carcinoma, 40% of the cases studied showing loss at this locus. We have made the conservative assumption that additional LOH demonstrated using markers for other loci on 11p and/or 11q indicate more general loss of a whole or hemichromosome 11, and therefore these cases have been excluded from further analysis. There is support for this assumption using data from RFLP deletion mapping studies (Srivatsan *et al.*, 1991) and from cytogenetic studies of cervical carcinoma which find such patterns of chromosomal 11 loss in between 10% and 30% of tumours (Atkin *et al.*, 1990; Sreekantaiah *et al.*, 1991). After exclusion of these cases, allelic loss at locus D11S29 occurred in 30% of cervical tumours in our hands.

Despite cytogenetic data showing chromosome 11 to be over-represented in structural aberrations in cervical malignancy, there has been little previous work looking at the molecular changes on chromosome 11. Srivatsan *et al.* (1991) using RFLP analysis on 15 cases of cervical carcinoma found a diversity of loci deleted in six tumour samples, the majority at sites on 11q, leading the authors to implicate the long arm as a putative location for a cervical cancer tumoursuppressor gene. Hampton *et al.* (1994) reported on an allelic mapping study of 32 cervical carcinomas using polymorphic markers, the majority being PCR-detected microsatellite polymorphisms on chromosome 11. This study found evidence of clonal LOH for one or more markers on 11q in 14 cases; the highest frequencies were observed with polymorphic loci at the D11S144 locus (52%) and the APOC3 gene (43%), both of which map to 11q23.

Foulkes *et al.* (1993), using microsatellite polymorphisms, produced a deletion map of chromosome 11q in 28 epithelial ovarian tumours. At the D11S29 locus they found LOH in 7 of 15 cases (47%). Interestingly, this study found a higher rate of LOH at adjacent more distal loci, implying that the area of maximum interest for a putative tumour-suppressor gene is distal to the CD3D locus on 11q23.3, which is some 1.8 cM distal from the D11S29 locus. 11q23 has also been of importance in haematological malignancies, with rearrangements of this band being very common in acute leukaemias. Here a number of translocations of the *MLL* gene (also known as the *ALL1* or *HRX* gene) from 11q23 lead to the creation of a fusion gene which encodes chimaeric oncogenic proteins (Rowley, 1993). The role of 11q23 in proto-oncogene activation in cervical malignancy remains to be investigated.

How the existence of a tumour-suppressor gene on human chromosome 11q fits into our current understanding of the aetiology of cervical carcinoma is uncertain. It is known that transfer of a single human chromosome 11 to either the HeLa (Saxon *et al.*, 1986) or SiHa (Koi *et al.*, 1989) cervical carcinoma cell lines suppresses the tumorigenic phenotype. It is now appreciated that several subtypes of human papillomavirus (HPV) occur frequently in cervical carcinoma and its precursor lesions, and that viral integration into host DNA affecting viral early (E) or late protein-encoding regions appears important in the oncogenic process (Lane and Wells, 1994). Compared with many other tumour types, inactivation of the p53 gene by allelic loss or mutation is an uncommon event in cervical carcinoma, however HPV E6 protein is known to bind to and neutralise normal p53 protein function. HPV integration sites have been shown to be located on chromosomes close to oncogenes, fragile sites and cancer chromosome breakpoints (Cannizzaro *et al.*, 1988), although chromosome 11 has not been implicated (De Braekeleer *et al.*, 1992). Interestingly, in experiments on human fibroblast cells co-transfected with an HPV 16 DNA containing plasmid with an activated EJ-*ras* oncogene, a translocation, t(1:11), was found to be a frequent event; the breakpoint was invariably at 11q23 (Matlashewski *et al.*, 1988). However, in the present series, LOH at D11S29 was not correlated with the presence of HPV as detected by PCR techniques. It is likely that HPV integration is a necessary but not sufficient precondition for cervical cancer, and there is a synergistic role for other oncogenes including c-*myc* and H-*ras* (Chen and Defendi, 1992). Loss or inactivation of putative tumour-suppressor genes on chromosomes 3p (Yokota *et al.*, 1989; Jones and Nakamura, 1992) and 17p (Russell *et al.*, 1992) has been reported in a proportion of cervical tumours, and the data from this and other studies suggest that allelic loss in the region 11q22–q24 may be implicated in the chain of malignant transformation in the cervix.

In summary, we have shown that loss of heterozygosity at the D11S29 locus on human chromosome 11q23 occurs in 40% of invasive cervical carcinomas. By excluding those cases where LOH is occurring secondarily to loss of a whole chromosome 11 or its long arm, then we find LOH at D11S29 in 30% of cervical carcinomas. Previous reports have shown LOH at this site in other tumour types (Stickland *et al.*, 1992; Foulkes *et al.*, 1993; Tomlinson *et al.*, 1993) with a high frequency of LOH at 11q22–q24 in small series of ovarian and cervical carcinomas (Foulkes *et al.*, 1993; Hampton *et al.*, 1994), implicating a candidate tumour-suppressor gene distally on chromosome 11q. The findings in the current study, while not providing a detailed deletion map of the 11q region, are consistent with the involvement of chromosome 11q in cervical carcinogenesis. Further work is now under way to produce a more detailed deletion map using other loci on chromosome 11q23 and distal sites in an attempt to pinpoint more accurately the region of interest. In addition, it is important to examine the pattern of loss in high- and low-grade cervical intraepithelial lesions to assess where events on chromosome 11 are acting in the chain of cervical malignant transformation.

**Acknowledgements**

Dr Bethwaite was funded by a research fellowship from the Worshipful Company of Girdlers, Dr Koreth by a Rhodes Scholarship. The work was also undertaken with partial financial assistance from the Cancer Research Campaign. The authors are especially grateful to Professor K Cooper, South African Institute For Medical Research, University of Witwatersrand, Johannesburg, who contributed a number of cases for this study. We also wish to thank Dr L Pan, University College London Medical School, for teaching us his microdissection technique and Mark Evans and Linda Bromley for their technical advice.

### References

ATKIN NB, BAKER MC AND FOX MF. (1990). Chromosome changes in 43 carcinomas of the cervix uteri. *Cancer Genet. Cytogenet.*, **44**, 229–241.

BAUER HM, GREER CE AND MANOS MM. (1992). Determination of genital human papillomavirus infection by consensus PCR amplification. In *Diagnostic Molecular Pathology: A Practical Approach*, Vol. II, Herrington CS and McGee JO'D. (eds). pp. 131–152. IRL Press: Oxford.

BETHWAITE PB, KORETH J, HERRINGTON CS AND MCGEE JO'D. (1994). Loss of heterozygosity occurs at the D11S29 locus on chromosome 11q23 in invasive cervical carcinoma (abstract). *J. Pathol.*, **173** (Suppl.), 196.

CANNIZZARO LA, DURST M, MENDEZ MJ, HECHT BK AND HECHT F. (1988). Regional chromosome localization of human papillomavirus integration sites near fragile sites, oncogenes and cancer chromosome breakpoints. *Cancer Genet. Cytogenet.*, **33**, 93–98.

CAWKWELL L, BELL SM, LEWIS FA, DIXON MF, TAYLOR GR AND QUIRKE P. (1993). Rapid detection of allele loss in colorectal tumours using microsatellites and fluorescent DNA technology. *Br. J. Cancer*, **67**, 1262–1267.

CHEN TM AND DEFENDI V. (1992). Function integration of p53 with HPV 8 E6, c myc, and H-ras in 3t3 cells. *Oncogene*, **7**, 1541–1547.

DE BRAEKELEER M, SREEKANTAIAH C AND HASS O. (1992). Herpes simplex virus and human papillomavirus sites correlate with chromosomal breakpoints in human cervical carcinoma. *Cancer Genet. Cytogenet.*, **59**, 135–137.

DON RH, COX PT, WAINWRIGHT BJ, BAKER K AND MATTICK JS. (1991). 'Touchdown' PCR to circumvent spurious priming during gene amplification. *Nucleic Acids Res.*, **19**, 4008.

FOULKES WD, CAMPBELL IG, STAMP GWH AND TROWSDALE J. (1993). Loss of heterozygosity and amplification on chromosome 11q in human ovarian cancer. *Br. J. Cancer*, **67**, 268–273.

HAMPTON GM, PENNY LA, BAERGEN RN, LARSON A, BREWER C, LIAO S, BUSBY-EARLE RMC, WILLIAMS AWR, STEEL CM, BIRD CC, STANBRIDGE EJ AND EVANS GA. (1994). Loss of heterozygosity in cervical carcinoma: subchromosomal localization of a putative tumor-suppressor gene to chromosome 11q22-q24. *Proc. Natl Acad. Sci. USA*, **91**, 6953–6957.

HAUGE XY, EVANS GA AND LITT M. (1991). Dinucleotide repeat polymorphism at the D11S534 locus. *Nucleic Acids Res.*, **19**, 4308.

JONES MH AND NAKAMURA Y. (1992). Deletion mapping of chromosome 3p in female genital tract malignancies using microsatellite polymorphisms. *Oncogene*, **7**, 1631–1634.

KOI M, HIROYUKI M, HIDETO Y, BARRET JC AND OSHIMURA M. (1989). Normal human chromosome 11 suppresses tumorigenicity of human cervical tumor cell line SiHa. *Mol. Carcinogen.*, **2**, 12–21.

LAKHANI SR, STRATTON MR, COLLINS N AND SLOANE JP. (1994). Detection of allele loss in preinvasive breast cancer suggests a high proportion of somatic genetic alterations are acquired before invasion (abstract). *J. Pathol.*, **172** (Suppl.), 17.

LANE S AND WELLS M. (1994). Human papillomavirus, p53 and cervical neoplasia. *J. Pathol.*, **172**, 299–300.

MATLASHEWSKI G, OSBORN K, BANKS L, STANLEY M AND CRAWFORD L. (1988). Transformation of primary human fibroblast cells with human papillomavirus type 16 DNA and EJ-*ras*. *Int. J. Cancer*, **42**, 232–238.

NORDENSKJOLD M. (1990). Recessive mutations in the oncogenesis of certain solid tumours. In *Tumour Suppressor Genes*, Klein G (ed.) pp. 145–168. Marcel Dekker: New York.

ORPHANOS V, MCGOWN G, BOYLE JM AND SANTIBANZE-KOREF MF. (1993). Thirteen dinucleotide repeat polymorphisms on chromosome 6. *Hum. Mol. Genet.*, **2**, 2196.

PAN LX, DISS TC, PENG HZ AND ISAACSON PG. (1994). Clonality analysis of defined B-cell populations in archival tissue sections using microdissection and the polymerase chain reaction. *Histopathol.*, **24**, 323–327.

PHROMCHOTIKUL T, BROWNE D AND LITT M. (1992). Microsatellite polymorphisms at the D11S554 and D11S569 loci. *Hum. Mol. Genet.*, **1**, 3.

ROWLEY JD. (1993). Rearrangements involving chromosome band 11q23 in acute leukemia. *Semin. Cancer Biol.*, **4**, 377–385.

RUSSEL SEH, LOWRY WS, ATKINSON RJ AND HICKEY I. (1992). Homozygosity of the short arm of chromosome 17 in cervical carcinoma. *Cancer Lett.*, **62**, 243–247.

SAXON PJ, SRIVATSAN ES AND STANBRIDGE EJ. (1986). Introduction of human chromosome 11 via microcell transfer controls tumourigenic expression of HeLa cells. *EMBO J.*, **5**, 3461–3466.

SOLOMON E, BARROW J AND GODDARD AD. (1991). Chromosome aberrations and cancer. *Science*, **254**, 1153–1160.

SREEKANTAIAH C, DE BRAEKELEER M AND HAAS O. (1991). Cytogenetic findings in cervical carcinoma: a statistical approach. *Cancer Genet. Cytogenet.*, **53**, 75–81.

SRIVATSAN ES, MISA BC, VENUGOPALAN M AND WILCZYNSKI SP. (1991). Loss of heterozygosity for alleles on chromosome 11 in cervical carcinoma. *Am. J. Hum. Genet.*, **49**, 868–877.

STICKLAND JE, TOMLINSON IPM, LEE ASG, EVANS MF AND MCGEE JO'D. (1992). Allele loss on chromosome 11q is a frequent event in breast cancer (abstract). *Br. J. Cancer*, **66** (Suppl. 17), 3.

TOMLINSON IPM, GAMMACK AJ, STICKLAND JE, MANN GJ, MACKIE RF, KEFFORD RF AND MCGEE JO'D. (1993). Loss of heterozygosity in malignant melanoma at loci on chromosome 11 and 17 implicated in the pathogenesis of other cancers. *Genes Chrom. Cancer*, **7**, 169–172.

WARNICH L, GROENEWALD I, THEART L AND RETIEF AE. (1992). Highly informative dinucleotide repeat polymorphism at the D11S29 locus on chromosome 11q23. *Hum. Genet.*, **89**, 357–359.

WHETSELL L, MAW G, NADON N, RINGER DP AND SCHAEFER FV. (1992). Polymerase chain reaction microanalysis of tumours from stained histological slides. *Oncogene*, **7**, 2355–2361.

YOKOTA J, TSUKADA Y, NAKAJIMA T, GOTOH M, SHIMOSATO Y, MORI N, TSUNOKAWA Y, SUGIMURA T AND TERADA M. (1989). Loss of heterozygosity on the short arm of chromosome 3 in carcinoma of the uterine cervix. *Cancer Res.*, **49**, 3598–3601.